# Stem diameter and rotational stability in revision total hip arthroplasty: a biomechanical analysis

**DOI:** 10.1186/1749-799X-1-5

**Published:** 2006-10-02

**Authors:** R Michael Meneghini, Nadim J Hallab, Richard A Berger, Joshua J Jacobs, Wayne G Paprosky, Aaron G Rosenberg

**Affiliations:** 1 Joint Replacement Surgeons of Indiana Research Foundation, St. Vincent Center for Joint Replacement, Indianapolis, IN, USA; 2 Department of Orthopaedic Surgery, Rush Medical College, Rush University Medical Center, Chicago, IL, USA

## Abstract

**Background:**

Proximal femoral bone loss during revision hip arthroplasty often requires bypassing the deficient metaphyseal bone to obtain distal fixation. The purpose of this study was to determine the effect of stem diameter and length of diaphyseal contact in achieving rotational stability in revision total hip arthroplasty.

**Methods:**

Twenty-four cadaveric femoral specimens were implanted with a fully porous-coated stem. Two different diameters were tested and the stems were implanted at multiple contact lengths without proximal bone support. Each specimen underwent torsional testing to failure and rotational micromotion was measured at the implant-bone interface.

**Results:**

The larger stem diameter demonstrated a greater torsional stability for a given length of cortical contact (p ≤ 0.05). Decreasing length of diaphyseal contact length was associated with less torsional stability. Torsional resistance was inconsistent at 2 cm of depth.

**Conclusion:**

Larger stem diameters frequently used in revisions may be associated with less diaphyseal contact length to achieve equivalent rotational stability compared to smaller diameter stems. Furthermore, a minimum of 3 cm or 4 cm of diaphyseal contact with a porous-coated stem should be achieved in proximal femoral bone deficiency and will likely be dependent on the stem diameter utilized at the time of surgery.

## Background

Proximal femoral bone loss during revision total hip arthroplasty is a common and challenging problem. Aseptic loosening and osteolysis may cause significant periprosthetic femoral bone destruction, often necessitating bypass of the deficient proximal femur to obtain stable fixation in the distal diaphysis [1-3]. The fixation should provide adequate initial implant stability to minimize micromotion and facilitate osseous ingrowth of the host bone into the prosthesis. In this setting of proximal bone loss, inadequate length of diaphyseal contact has been shown to correlate with a high clinical failure rate [1]. As a consequence, a minimum of 4 cm to 6 cm of diaphyseal contact length has been recommended and is associated with improved clinical results and a lower failure rate [1].

Clinical and biomechanical studies suggest that clinical failure of the femoral component is likely due to torsional forces applied to the prosthesis [4-8]. Femoral construct properties that may affect torsional stability include stem diameter, surface finish, interference fit and length of diaphyseal contact. Porous coating provides a rough surface for frictional resistance as well as an excellent surface for bone ingrowth. Maximizing the surface area of porous coating in contact with diaphyseal cortical bone has been shown to decrease implant micromotion and promote osseointegration [9]. Theoretically, implant surface area in contact with cortical bone may then be increased either by increasing the length of diaphyseal contact or by increasing the stem diameter and subsequent circumference of the stem surface. These mechanical factors, as well as biological conditions, determine the initial femoral component resistance to torsional loads. Optimizing these factors provides the mechanical stability necessary for osseous integration and subsequent long-term success of the femoral implant.

Various studies have investigated the torsional stability of cemented and cementless femoral stems with regard to implant design, distal fixation characteristics, reaming technique and surgical press-fit technique [4,10-18]. However, the authors are not aware of any study which specifically investigates the effect of stem diameter on achieving rotational stability in the revision setting. Furthermore, little data exists on the actual length of diaphyseal contact necessary to obtain implant stability in the setting of proximal femoral bone deficiency. The purpose of this study was to determine the effect of stem diameter on torsional stability in a biomechanical analysis of cadaveric femurs, as well as investigate the length of cortical contact necessary to obtain sufficient torsional stability for osseointegration.

## Methods

**Figure 1:**
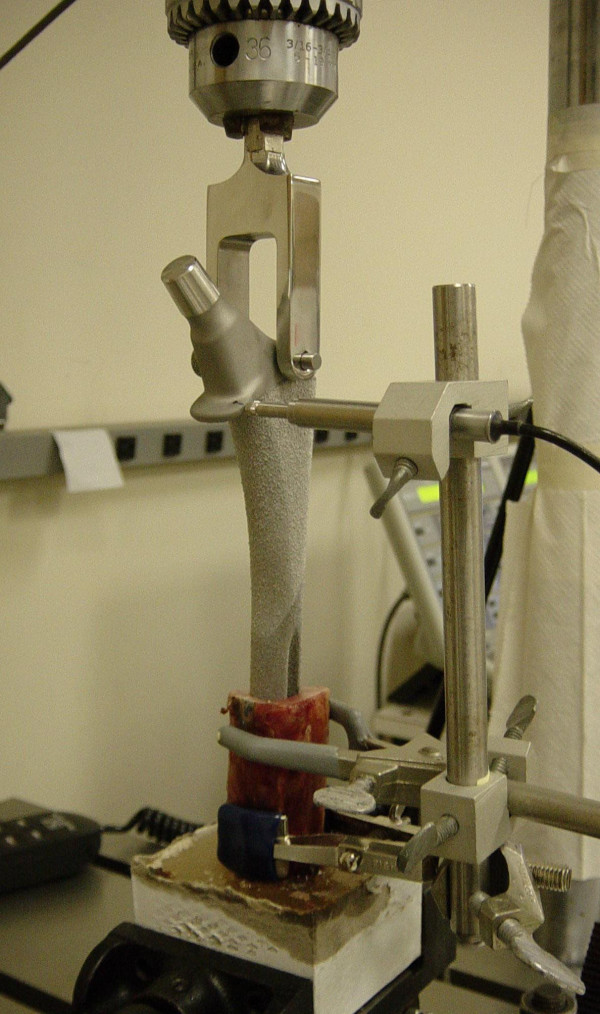
Instron testing machine setup with load cell attached to implanted femoral component. LVDT is seated on widest part of the femoral component flange.

The femoral component utilized in this study is a straight, uncemented, cylindrical, fully porous-coated implant (Beaded Fullcoat Plus; Zimmer, Warsaw, IN), [Fig 1]. The stem diameters of 15 mm or greater are manufactured with distal flutes to minimize the bending stiffness associated with larger sizes. Two stem diameters, 15 mm and 18 mm, were chosen for testing in order to eliminate the confounding variable introduced by the differing cross-sectional geometry of the smaller diameter implants.

Thirty-two fresh-frozen human anatomic femora (sixteen matched pairs) were selected for testing. All specimens underwent visual inspection in addition to plain film radiography to ensure there were no cortical diaphyseal defects. The bone quality of each specimen was graded radiographically by Dorr's classification [19]. All specimens tested were graded as either type A or B. Two specimens were discarded due to extremely poor bone quality (type C) and with the canal size greater than 18 mm.

All femoral specimens were prepared in an identical manner. The same surgeon implanted all components in order to minimize variability associated with the implantation technique. The proximal femur was resected just below the metaphyseal-diaphyseal junction. The remaining diaphyseal segment was cleaned of all loose tissues and potted in acrylic cement to a minimum depth of 3 cm. Progressively larger straight reamers were used to enlarge the canal and create a uniform and parallel surgical isthmus. The canal was undersized by 0.5 mm to create a press-fit of the femoral component into the canal. The exact size of each femoral canal, straight reamer and femoral stem were confirmed with digital calipers for each specimen. The femoral component was inserted with manual impaction to the desired diaphyseal depth. Six femoral specimens sustained a fracture during stem impaction and were discarded. Anteroposterior roentgenograms of each femoral specimen with the implanted component were obtained prior to testing to ensure direct contact with the isthmus over the desired diaphyseal depth.

Each specimen was mounted in an Instron servohydraulic testing machine (Model 1321, Instron, Canton, Massachusetts) so the long axis of the femoral stem, the rotational axis of the Instron machine and the femoral specimen were collinear. A linearly variable differential transducer (LVDT; S5, Honeywell Sensotec, Columbus, OH) with a linear range of 2.5 mm and a repeatability of 0.5 μm was utilized to detect rotational micromotion. The LVDT was mounted on a clamp attached securely to the outer cortex of the cadaveric specimen and the LVDT sensor seated perpendicular to the widest portion of the prosthesis collar [Figure 1]. Similar experimental setups, utilizing LVDT measurement of rotational micromotion, have been well documented and accepted in the orthopaedic literature [4,10,11,15]. The torque load cell output and LVDT output were sampled at a frequency of 50 Hz and recorded in real time using a computerized data acquisition system (FastTrack2, Instron, Canton, Massachusetts). Linear LVDT measurements were trigonometrically converted to rotational micromotion at the implant-bone interface using the known distance from the LVDT contact point to the stem center of rotation and the stem radius.

A torque load was applied to each specimen under displacement-control at an angular rate of 0.5° per second. A constant axial load of 700 N was applied to the implant throughout the torsional testing to simulate weight bearing. A 5 Nm torque preload was applied to each specimen and maintained for 5 seconds. Upon completion of the preload, the test was initiated at 1 Nm of torque and carried out until torsional failure. Torsional failure was defined as either fracture of the bone, 150 μm of rotational micromotion or an abrupt change in the slope of the torque-displacement curve. Twenty-four specimens underwent torsional testing to failure. The femoral implants of two diameters (15 mm and 18 mm) were subjected to torsional loads at each of the three diaphyseal contact lengths (4 cm, 3 cm and 2 cm), yielding six groups of four specimens in each group [Table 1]. The load cell output and LVDT output converted to interface micromotion generated a torque-displacement curve in each test. Studies have shown that implant micromotion in the range of 40 μm to 150 μm typically provides sufficient stability for osseous integration [9,20-22]. Therefore, the torque resistance measured at 40, 50, 100 and 150 micrometers (μm) of rotational micromotion was considered clinically relevant and was recorded for each specimen.

**Table 1:** Results of mean torsional resistance for studied stem diameters and diaphyseal contact depths. Group Mean Torsional Resistance Data *

**Size 18:**		**40 um**	**50 um**	**100 um**	**150 um**	**ε**
**4 cm**	**Mean:**	**18.94**	**21.48**	**29.56**	**32.32**	**0.3972**
	SD:	2.51	1.97	5.17	8.02	0.0939
**3 cm**	**Mean:**	**16.87**	**19.96**	**26.21**	**25.8**	**0.3398**
	SD:	1.26	2.49	5.15	5.82	0.0695
**2 cm**	**Mean:**	**11.39**	**13.04**	**20.31**	**23.02**	**0.2532**
	SD:	7.42	9.16	15.38	15.54	0.1666

**Size 15:**		**40 um**	**50 um**	**100 um**	**150 um**	**ε**

**4 cm**	**Mean:**	**13.24**	**15.43**	**21.54**	**23.2**	**0.2378**
	SD:	2.67	2.85	4.34	6.14	0.0741
**3 cm**	**Mean:**	**10.69**	**13.26**	**23.41**	**27.49**	**0.258**
	SD:	3.36	4.5	8.13	7.16	0.086
**2 cm**	**Mean:**	**7**	**8.07**	**13.07**	**17.66**	**0.0958**
	SD:	1.58	1.79	2.34	3.16	0.0242

* all units are Newton-meters (Nm) except interface stiffness

**ε **= interface stiffness (um/Nm)

The slope of the linear portion of the torque-displacement curves was calculated using linear regression analysis. The slope is considered the *interface stiffness *(ε) of the bone-prosthesis interface. A Pearson correlation coefficient was calculated for each slope value to assess the strength of that linear relationship. The unpaired Student t-test was used to compare differences in mean torque resistance between stem sizes (15 mm and 18 mm) at each of the diaphyseal depths (2, 3, and 4 cm). One-way analysis of variance (ANOVA) was used to compare differences in torque resistance across the three diaphyseal depths for each stem size. The LSD post-hoc test was used when the F test was significant. A factorial ANOVA was used to examine the interaction effect between stem size and diaphyseal contact length for torque resistance at 40 μm, 50 μm, 100 μm and 150 μm of rotational micromotion. A significance level of less than 0.05 was considered statistically significant.

## Results

**Figure 2:**
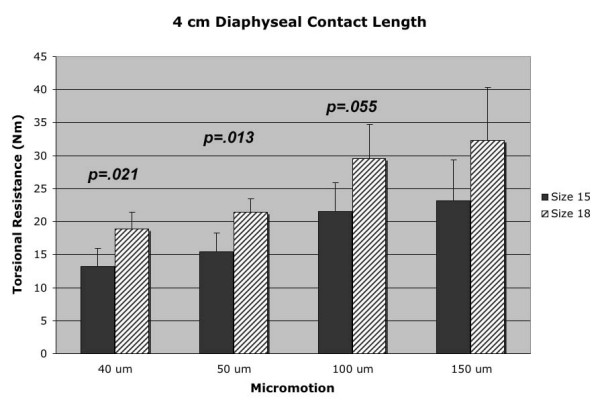
4 cm diaphyseal contact length (depth) data for both 18 mm and 15 mm diameter stems at the four points of measured rotational micromotion.

The results demonstrated greater mean torsional resistance for the larger 18 mm diameter stem, when compared to the smaller 15 mm stem, at the various measured points of rotational micromotion for a given diaphyseal depth [Table 1]. Figure 2 shows the mean torsional resistance data for the 4 cm diaphyseal contact length (depth) at 40 μm, 50 μm, 100 μm and 150 μm of micromotion. The larger 18 mm stem diameter group demonstrated significantly greater torsional resistance at the 40 μm (p = 0.021) and 50 μm (p = 0.013) interface micromotion points, when compared to the 15 mm stem diameter group at the 4 cm diaphyseal contact length. In addition, the 18 mm stem group demonstrated greater torsional resistance at the 100 μm micromotion point over the 15 mm stem that was very close to reaching statistical significance (p = 0.055).

**Figure 3:**
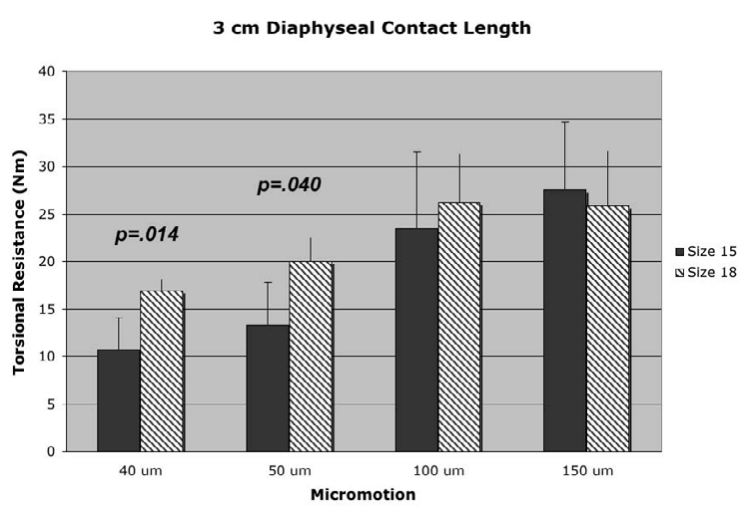
3 cm diaphyseal contact length (depth) data for both 18 mm and 15 mm diameter stems at the four points of measured rotational micromotion.

**Figure 4:**
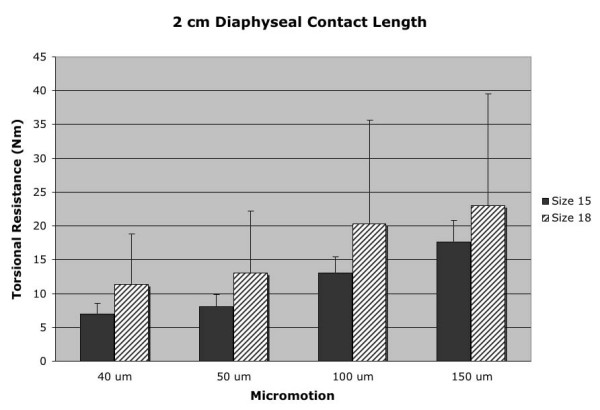
2 cm diaphyseal contact length (depth) data for both 18 mm and 15 mm diameter stems at the four points of measured rotational micromotion.

Mean torsional resistance data for the 3 cm diaphyseal contact length test groups is represented in Figure 3. The larger 18 mm diameter stem demonstrated an increase in torsional resistance with statistical significance at the 40 μm (p = 0.014) and 50 μm (p = 0.040) micromotion points. A statistically significant difference was not demonstrated at any micromotion point at the 2 cm diaphyseal depth, despite the larger group means for torsional resistance of the 18 mm diameter stem over the smaller 15 mm stem [Figure 4, Table 1]. The lack of statistical significance at the 2 cm diaphyseal depth is likely related to the large standard deviations of the 18 mm diameter stems tested at this diaphyseal contact length.

**Figure 5:**
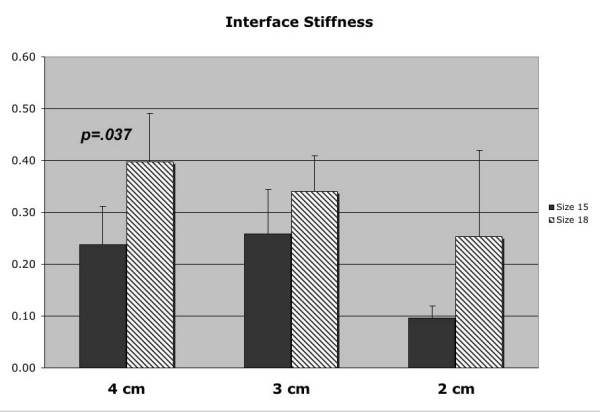
Interface stiffness (ε) data for both 18 mm and 15 mm diameter stems at the various diaphyseal contact lengths.

*Interface stiffness *(ε), as determined by the slope of the linear portion of the torque-displacement curve, was greater for the 18 mm diameter stems than those values for the 15 mm stem at each diaphyseal contact length [Figure 5, Table 1]. However, only the 4 cm diaphyseal depth demonstrated a statistically significant difference (p = 0.037) in the mean *interface stiffness *(ε) between 18 mm and 15 mm diameter stems. All specimen interface stiffness data demonstrated linear behavior prior to failure, with correlation coefficient values of greater than 0.98 with linear regression analysis.

The torsional resistance at the measured points of micromotion was compared within each stem size, among the different diaphyseal contact lengths. The 18 mm diameter stem demonstrated greater torsional resistance values and interface stiffness (ε) with increasing diaphyseal depth; however, no statistically significant difference (p > 0.05) was found when compared at 4 cm, 3 cm or 2 cm of diaphyseal contact length. In contrast, the 15 cm diameter stem demonstrated greater mean torsional resistance at the 4 cm diaphyseal contact length when compared to the 2 cm diaphyseal contact length at 40 μm (p = 0.007), 50 μm (p = 0.005) and 100 μm (p = 0.014). In addition, the 15 mm diameter stem exhibited greater torsional resistance for the 3 cm contact length when compared to the 2 cm depth at 100 μm (p = 0.050) and 150 μm (p = 0.046) of micromotion. Moreover, the difference in interface stiffness (ε) among the various contact depths of the 15 cm stem reached statistical significance when comparing 4 cm versus 2 cm (p = 0.011) and 3 cm versus 2 cm (p = 0.011) depths.

## Discussion

In the setting of proximal femoral bone loss, obtaining adequate distal diaphyseal fixation is essential in revision total hip arthroplasty with cementless porous-coated femoral implants. There is little data regarding the effect of femoral component diameter on achieving rotational stability in the revision setting. Furthermore, the length of diaphyseal contact and type of implant necessary to optimize implant fixation and biologic ingrowth has not been conclusively determined. Our understanding of bypass fixation in the periprosthetic femur with deficient bone stock has come largely from studies involving femoral component fixation with cement. Two retrospective outcome studies of cemented revision total hip arthroplasty recommended bypassing femoral cortical defects by a minimum of two femoral shaft diameters [23,24]. Biomechanical studies with cemented stems recommended bypassing cortical defects by one to two femoral diameters [5,25]. However, despite these clinical and biomechanical studies, cement fixation of the revision stem is associated with decreased bone-cement interface shear strength [26], as well as high re-revision rates for aseptic loosening [23,24]. These clinical and biomechanical studies using cemented implants are not likely applicable to implant stability with cementless porous-coated stems.

Long-term biologic fixation has been shown to be obtainable via extensively porous-coated stems, even in the face of proximal femoral deficiency [1,3]. In a retrospective review of revision hip arthroplasty using extensively porous-coated stems, Paprosky et al reported a survivorship of greater than 95% and a low 4.1% failure rate at a minimum of ten-year follow-up. However, a femoral component failure rate of 21 percent was noted in femurs with less than 4 cm of diaphyseal contact. The authors recommended a minimum of 4 cm diaphyseal contact with adequate canal fill to obtain appropriate implant stability [1]. Furthermore, Engh et al reported their long-term results of revision total hip arthroplasty with severe proximal femoral bone loss extending at least 10 cm distal to the lesser trochanter. The authors reported adequate results when bypassing the deficient bone with extensively porous-coated implants, with a survivorship of 89 percent at ten years [3].

There are numerous biomechanical studies in the current literature regarding torsional stability of cementless femoral components [4,7,10-16,18]. These studies employ a variety of experimental protocols and loading conditions and have analyzed a multitude of variables including cemented versus uncemented fixation, proximal and distal fixation, reaming technique and implant design. However, to our knowledge, there are no biomechanical studies that have specifically addressed isolated stem diameter and diaphyseal contact length with regard to torsional stability in proximal femoral deficiency. The effect of femoral component press-fit on torsional fixation was studied in a biomechanical analysis [15]. The authors reported superior rotational stability of the femoral implant when the diaphysis was under-reamed by 0.5 mm when compared to line-to-line reaming. However, the femoral components were implanted into femoral specimens with retention of the proximal metaphysis, incorporating proximal fixation into the biomechanical testing [15]. In another biomechanical study, authors reported inferior torsional stability in isolated distal diaphyseal fixation when compared to specimens with both proximal and distal fixation [10]. In the same study, cementless porous-coated femoral stems of two different lengths were inserted into cadaveric femoral specimens after removal of the proximal portion. Biomechanical testing demonstrated an increase in torsional stability with both increased diaphyseal contact length and increased direct contact area. The authors recommended 10 mm to 40 mm of tight, under-reamed, diaphyseal contact length to obtain sufficient torsional stability in the absence of proximal bone stock [10]. In the only biomechanical study to address the issue of stem diameter, no correlation was found between torsional loosening loads of cementless components and stem size (13.5 mm and 15 mm). However, the proximal femur was retained in all specimens, employing both proximal and distal fixation into the biomechanical data [16]. Micromotion is likely directly related to the extent of porous coating on the implant [9]. In addition, increased torsional resistance has been observed with increased diaphyseal contact length and contact area in a cadaveric femur study using porous-coated femoral components [10].

The current study was undertaken to test our hypothesis that larger femoral stems demonstrate greater torsional stability in the setting of isolated diaphyseal fixation. Due to an increase in circumference, larger diameter cylindrical stems will theoretically have a greater surface contact area over a given length of femoral diaphysis, resulting in greater torsional stability. Our findings support this hypothesis with statistical significance (p < 0.05) at multiple levels of rotational micromotion, tested at both 4 cm and 3 cm of diaphyseal contact length. At 4 cm and 3 cm of diaphyseal contact, the mean torsional resistance of the larger 18 mm diameter stem was greater than the 15 mm diameter stem at multiple levels of measured rotational micromotion. In addition, greater interface stiffness (ε) at the porous-coated implant surface and the diaphyseal bone was demonstrated for the larger 18 mm diameter stem at all three measured contact lengths and reached statistical significance (p = 0.027) for the 4 cm diaphyseal depth [Figure 6]. Therefore, in the setting of severe proximal bone loss, larger stem diameters may provide greater implant stability against torsional loads due to the increase in contact area of the porous coating.

The 18 mm diameter stem demonstrated a wide variability in torsional stability at the minimal 2 cm diaphyseal contact length as indicated by large standard deviations in mean torsional resistance values [Table 1, Figure 5]. It has been recommended that 10 to 40 mm of intimate diaphyseal contact be obtained in the setting of absent or deficient femoral bone based on cadaveric studies [10]. However, based on the results obtained in this biomechanical analysis, a scratch-fit of 2 cm or less should be avoided in this clinical situation.

Despite these correlative results between stem sizes and diaphyseal contact length, the absolute torsional resistance values obtained in this study may be inadequate against the peak in vivo torsional loads experienced during activities such as walking and stair climbing. In a report on in vivo torsional loads via a telemeterized total hip component, a peak torque load of 23 Nm was observed in a patient during stair ascent without any assisting device [27]. The majority of the reported torsional resistance values for the lower levels of micromotion (40 μm and 50 μm) obtained in this study are below the peak loads reported to occur in vivo. This discrepancy has also been reported in other cadaveric biomechanical studies of isolated distal fixation [10,15,18], highlighting the difficulty of obtaining torsional stability in the setting of severe proximal bone loss. Therefore, it is likely that proximal femoral bone contributes clinically to the overall torsional stability of the femoral construct and in the absence of this proximal support, the authors recommend maintaining a minimum of 3 cm to 4 cm of diaphyseal contact. Further research is warranted to ascertain whether other implant designs, such as fluted, tapered, modular stems, may achieve improved clinical success in this difficult setting.

There are limitations in this study. These results are obtained using mechanical simulation in cadaveric femora and fail to account for the effects of biological osseous ingrowth over time. Furthermore, facility limitations prohibited the use of more accurate measures, such as bone densitometry, to assess cadaveric bone quality, which certainly plays a role in the torsional stability of press-fit cementless implants. In addition, there is no consensus as to the most accurate method of simulating the biomechanical loading conditions experienced by the femoral component in situ. Therefore, additional biomechanical studies using a greater range of sizes and loading regimens should be performed. Results from these biomechanical studies should be carefully correlated with long-term clinical outcomes in order to more accurately address the difficult issue of obtaining isolated diaphyseal fixation when bypassing deficient femoral bone stock. Currently, we recommend that diaphyseal contact length should be maximized to the extent that is technically possible in order to optimize femoral component stability in revision total hip arthroplasty. However, this study provides useful information pertaining to the role of femoral stem diameter and diaphyseal contact length in the tenuous clinical scenario where available diaphyseal fixation is limited.

## Conclusion

In summary, when obtaining diaphyseal bypass fixation of severe proximal bone deficiency, torsional stability of porous-coated femoral implants is related to the length of diaphyseal contact in addition to the stem diameter. Larger diameter femoral implants achieve greater torsional stability when compared to smaller stems at a given diaphyseal contact length. Therefore, this data suggests that when using a stem of larger femoral diameter where adequate diaphyseal contact can be reliably achieved, the surgeon may accept less diaphyseal contact than would be allowed for a smaller diameter stem to maintain sufficient torsional stability for clinical success. In this study, 2 cm of diaphyseal contact length was associated with both inadequate torsional resistance in the smaller diameter stems and a high degree of variability in the larger stems. Therefore, a minimum diaphyseal contact length of 3 cm or 4 cm is recommended to achieve adequate rotational stability with fully coated stems in revision total hip arthroplasty with proximal femoral bone loss.

## Competing interests

The author(s) declare that they have no competing interests.

## Authors' contributions

RMM designed the investigation protocol, performed all laboratory testing and data acquisition and coordinated and directed the manuscript preparation. NJH assisted in the development of the investigation protocol, assisted with all laboratory testing and data acquisition and assisted in drafting the manuscript. RAB conceived the study, assisted with development of the investigation protocol and assisted with drafting the manuscript. JJJ, WGP and AGR participated in the investigation concept and design, as well as assisted with manuscript preparation and drafting. All authors have read and approved the final manuscript.
